# Ethnoveterinary treatments for common cattle diseases in four districts of the Southern Province, Zambia

**DOI:** 10.14202/vetworld.2018.141-145

**Published:** 2018-02-08

**Authors:** Michelo Syakalima, Martin Simuunza, Victor Chisha Zulu

**Affiliations:** 1 Department of Animal Health, School of Agricultural Sciences, Faculty of Natural and Agricultural Sciences, North-West University, Mafikeng Campus, South Africa; 2 Department of Disease Control, School of Veterinary Medicine, Great East Road Campus, Box 32379. Lusaka, Zambia; 3 Department of Clinical Studies, School of Veterinary Medicine, Great Ease Campus, Box 32379, Lusaka, Zambia

**Keywords:** cattle, ethnomedicines, traditional farmers, Zambia

## Abstract

**Aim:**

Ethno veterinary knowledge has rarely been recorded, and no or limited effort has been made to exploit this knowledge despite its widespread use in Zambia. This study documented the types of plants used to treat important animal diseases in rural Zambia as a way of initiating their sustained documentation and scientific validation.

**Materials and Methods:**

The study was done in selected districts of the Southern Zambia, Africa. The research was a participatory epidemiological study conducted in two phases. The first phase was a pre-study exploratory rapid rural appraisal conducted to familiarize the researchers with the study areas, and the second phase was a participatory rural appraisal to help gather the data. The frequency index was used to rank the commonly mentioned treatments.

**Results:**

A number of diseases and traditional treatments were listed with the help of local veterinarians. Diseases included: Corridor disease (Theileriosis), foot and mouth disease, blackleg, bloody diarrhea, lumpy skin disease, fainting, mange, blindness, coughing, bloat, worms, cobra snakebite, hemorrhagic septicemia, and transmissible venereal tumors. The plant preparations were in most diseases given to the livestock orally (as a drench). Leaves, barks, and roots were generally used depending on the plant type.

**Conclusion:**

Ethno veterinary medicine is still widespread among the rural farmers in the province and in Zambia in general. Some medicines are commonly used across diseases probably because they have a wide spectrum of action. These medicines should, therefore, be validated for use in conventional livestock healthcare systems in the country to reduce the cost of treatments.

## Introduction

Rural farmers in most parts of Africa are a rich source of practical livestock husbandry and animal health information [1,2]. The quality and depth of this knowledge usually correspond to the degree of the community’s economic dependence on livestock and the prevailing diseases in that country [3-5]. The animal health knowledge will usually include assigning local names to a number of livestock diseases as well as describing the causes of the diseases to cover what conventional medicine classifies as etiology, clinical, and pathological signs [6]. Furthermore, these livestock farmers develop strategies to control and successfully treat diseases using existing local knowledge [7]. This invaluable knowledge has helped these communities keep livestock for generations even in the face of serious disease outbreaks [1,2].

People of Southern Province of Zambia are agro-pastoralists rearing about 50% of the country’s cattle population [8]. For generations, these livestock keepers have been using both indigenous knowledge and conventional medicines to safeguard the health of their livestock. Regrettably, this local knowledge has seldom been recorded, and no effort has been made to exploit this know-how when designing herd health and production strategies. Using such strategies would make treatment and control affordable and sustainable as has been observed in other countries [9,10]. Furthermore, as in most African societies, this knowledge is only passed from one generation to another as an inheritance within family lines and is never or rarely made available for public use [11]. Sometimes, it is fully owned by specific members of society to whom people will go and consult and thus not very much of it is in public domain [12,13]. Although about 80% of Africans depend on traditional medicines, most of this will be toward human and not animal diseases as has been noticed in a study in South Africa [14]. This deters the role of ethnoveterinary medicines in animal health care and in livestock-based economies.

The purpose of this study was to document Zambian traditional farmers’ knowledge of what they consider to be the most important diseases of their livestock and the ethnoveterinary treatments they use against these diseases. To the authors’ knowledge, this is the first effort of its kind to document ethnoveterinary plants used in the province and the country as a whole. This study will hopefully stimulate further work on the validation of these plants pharmacologically.

## Materials and Methods

### Ethical approval

This research was approved by the School of Veterinary Medicine research committee and approval for the meetings was sought from community leaders. Furthermore, consent was sought from all knowledge givers and they were told that they could only give information voluntarily and that when they declined there were no consequences.

### Study sites

**Figure-1:**
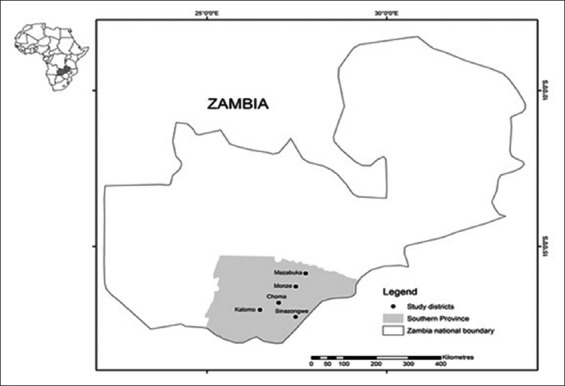
Map showing the study sites in the Southern Province of Zambia, Africa.

The study was done in Choma, Kalomo, Monze, and Sinazong we districts of the Southern province of Zambia, Southern Africa (Figure-1).

### Study plan

The research was a participatory epidemiological study conducted in two phases. The first phase was a pre-study exploratory rapid rural appraisal conducted to familiarize the researchers with the study areas, learn the social structure of the communities, type of livestock prevalent, and identification of key informants and entry points. This was important for getting the local people’s consent and cooperation.

The second phase was a participatory rural appraisal (PRA). The PRA methodology facilitated a rapid overview of animal health problems in the study areas and treatment and control strategies applied using an open-ended questionnaire. This was achieved by directly asking livestock owners animal health problems that were occurring or had occurred and treatments used. Focus group discussions (FGDs) were used to build a definition of each livestock problem. Once the description was received, clarifying questions were asked. After a number of interviews had been completed, consensus definitions (in vernacular) of important animal health diseases were determined. The diseases, their diagnoses, and treatments were further verified with local veterinarians and their records. After the FGDs in-depth interviews were done with key informants identified from the group to further validate the information. In each district where the study was implemented informants were used to identify the key knowledge holders who would verify the treatments. Only knowledge holders who were willing to participate were engaged after they understood and consented to the study.

### Data recording

The responses from the discussions during the PRAs were written down in notebooks as well as tape recorded. Tape recording was important so that where written notes were not well taken; they could be reviewed latter from the recordings. The four most frequently and commonly mentioned treatments, in order1of mention where possible, were noted for each disease. The frequency was calculated by way of a frequency index which is a numerical expression of the percentage frequency of citation for a single plant species by informants [15]. The following formula was used to calculate frequency index:

FI ¼ FC=N 100

Where FC is the number of informants, who mentioned the use of the plant species and N is the total number of informants in each area. In each district where the study was implemented 60 people (i.e., 30 per study site×2 sites) participated, giving a total sample size of 240 in the whole study. The 30 people per site were the minimum number of people belonging to each of the farmers’ cooperative at each site as suggested by the cooperative leadership. The frequency index was high when there were many informants that mentioned a particular plant and low when there were few reports.

## Results

### List of diseases and their treatments

Table-1 summarizes the common diseases of cattle mentioned by the local farmers and verified by local veterinarians and the plants used to treat them in the four districts of Southern Province, Zambia.

**Table-1:** Plants used to treat against the listed diseases common in cattle of the Southern Province of Zambia.

Disease/clinical sign	Botanical name (vernacular/local name) (frequency index)	Plant part	Preparation and route
Corridor Disease (Theileriosis)	*Cassia Abbreviata/Singueana* or *Trema* *Orientalis* (Mululwe) (97.9), *Azadirachta indica* (Neem tree) (85.4) *Colophospermum* *Mopane* (Mwaani), (66.6) *Julbernardia* *Globiflora* (Muumba) (58.3)	Roots and the bark Leaves Bark Roots	Concoction given as a drench
Foot and Mouth Disease	*Cassia Abbreviata*/*Singueana Trema* *Orientalis* (Mululwe), (79.2) *Colophospermum* *Mopane* (Mwaani) (58.3) *Cissus quadrangularis* (Namununga) (57.5)	Roots and Bark Bark Roots	Concoction given as a drench
Black Leg	*Cassia Abbreviata*/*Singueana Trema* *Orientalis* (Mululwe) (45.8)	Roots and bark	Concoction given as a drench
Bloody Diarrhea	*Cassia Abbreviata*/*Singueana Trema* *Orientalis* (Mululwe), (99.1) *Julbernardia* *Globiflora* (Muumba) (93.7) *Cissus quadrangularis* (Namununga) (88.3)	Roots and bark Roots Roots	Concoction given as a drench
Lumpy Skin Disease	*Cassia Abbreviata*/*Singueana Trema* *Orientalis* (Mululwe) (37.5) *Apply used oil on the skin* (98.3)	Roots and bark	Concoction given as a drench or applied to the skin
Fainting	*Steganotaenia Araliacea* (Mpe lefu/Mutobolo) (50)	Roots	Concoction given as a drench
Mange	*Julbernardia* *Globiflora* (Muumba) (54.1)	Bark	Applied to the skin
Blindness	*Strychnos Potatorum* (Musisi/musisi) (66.3)	Leaves	Given as eye drops
Coughing	*Cassia Abbreviata*/*Singueana Trema* *Orientalis* (Mululwe) (59.6) *Cissus quadrangularis* (Namununga) (44.5)	Roots and the bark Roots	Concoction given as a drench
Bloat	*Cassia Abbreviata*/*Singueana Trema* *Orientalis* (Mululwe) (46.3)	Roots and bark	Concoction given as a drench
Worms	*Cissus* *quadrangularis* (Namununga) (88.8) *Cassia Abbreviata*/Singueana Trema *Orientalis* (Mululwe) (47.5)	Roots Roots and bark	Concoction given as a drench
Cobra Snake bite	*Piliostigma* *thonningii* (Musekese) (96.3) *Strychnos spinosa* (Maabo) (83.3)	Roots and bark Leaves	Concoction given as a drench And also applied to the bite site
Hemorrhagic Septicemia	*Julbernardia* *Globiflora* (Muumba) (58.3) *Cassia Abbreviata*/*Singueana Trema* *Orientalis* (Mululwe), (40)	Roots Roots and bark	Concoction given as a drench
Transmissible venereal tumor	*Albizia Harveyi* (Mukangala) (33.8)	Roots	Applied directly to the tumor site

### General preparation of the herbal medicine

The plant preparations were in most diseases given to the livestock orally (as a drench). Leaves, barks, and roots were generally used depending on the plant. These plant parts were pounded, crushed or in an intact form soaked in cold water or boiled, cooled and then administered as a drench. In some cases, a little salt or milk was added to the preparation before drenching the animals.

For cutaneous lesions such as a wound, the leaves, barks, and roots of plants were pounded or crushed and a little water added then applied directly to the skin condition or wound site.

In case of ophthalmic lesions the leaves, barks, and/or roots of these plants were pounded or crushed, water was added to the preparation, and it was then sieved through a cloth and applied directly to the eye.

## Discussion

It was apparent from this study that the use of ethno veterinary medicine is still widespread among the rural farmers in the province. All farmers interviewed indicated that they often used these treatments because they were readily available, always effective and less costly. This is an important aspect which has come out in a previous study [2] and underlines the key role these medicines have on veterinary health delivery systems in poor communities of Africa. Documenting and validating these ethno veterinary medicines should, therefore, be encouraged.

Some medicines such as Mululwe (*Cassia abbreviate/singueana* or *Trema orientalis*) were commonly mentioned by these farmers on many diseases at different study sites. The frequency by which some of these medicines were mentioned may to a certain extent validate their effectiveness on the diseases mentioned. Analyzing the complete chemical composition as well as the spectrum of action is there important to validate their putative efficacy in many conditions. A review of previous studies conducted by Mongalo and Mafoko [16] showed that *C. abbreviate* contains anthocyanins, anthranoids, anthraquinones, polyphenols, and tannins which have been associated with antibacterial, antifungal, antimalarial, anthelmintic, antiviral, antioxidant, and antidiabetic activities. This may explain why in our study, the plant is used in a number of conditions where the activities reviewed above may be required. As for *Trema orientalis*, a review by Adinortey *et al*. [17], revealed hypoglycemic, analgesic, anti-inflammatory, antiplasmodial, diuretic, laxative effect, anticonvulsant, anthelmintic, antisickling effect, antioxidant, and antibacterial activities. It is, therefore, possible that the use of these plants by these local farmers may be related to these reviewed effects. However, more studies are required.

Some conditions such as snake bites were found to be well understood and effectively treated by traditional farmers. In conventional veterinary practice, a snakebite is one of the most difficult treatments to carry out because the exact type of snake involved should be known before the right antivenom can be decided on [18]. This is usually not possible because the bites take place in the bush and the snake involved would rarely be known. Fortunately, the ethno veterinary treatments rarely require knowing the type of snake involved for the treatment to be effective. The treatments mentioned can be used across all the different snakebites thus very ideal for use in such conditions.

## Conclusion

In Zambia ethno veterinary knowledge has almost never been recorded despite widespread use by most rural communities. There has also been no particular interest by government and other stakeholders to exploit this knowledge in different animal health programs being offered. This is a serious oversight because this knowledge if properly harnessed could prove cheaper, sustainable, and widely effective. This study is therefore meant to initiate the wider documentation and validation of these plants in order for their better understanding and exploitation in animal health.

## Authors’ Contributions

MaS led the study design, field data collections and revised the manuscript. MaS was involved with data collection, analysis, and manuscript writing. VCZ was also involved in data collection and manuscript writing. All the authors read and approved the final manuscript.
